# Topical ivermectin 10 mg/g cream alone or in combination with oral doxycycline for patients with perioral dermatitis (POD): A retrospective case series

**DOI:** 10.1111/ddg.15691

**Published:** 2025-03-22

**Authors:** Martin Schaller, Daniela Lenders, Gabi Handgretinger, Andrea Gawaz

**Affiliations:** ^1^ Department of Dermatology Eberhard Karls University Tübingen Germany

Dear Editors,

Perioral dermatitis (POD), also known as rosacea‐like dermatitis, is a relatively common inflammatory chronic facial skin disorder.^1^, ^2^, ^3^ It is characterized by stinging, burning, itching and often bilateral erythematous papulovesicles or papulopustules that may stay over weeks to months. Although the perioral area is the most common site, it can also affect the periocular and paranasal skin. Most patients (approx. 90% of cases) are women between the ages of 20 and 45 years.^4^, ^5^

The etiology remains unclear, but it is thought to be multifactorial. The role of topical steroids as a trigger of rosacea is well established. Besides that, exaggerated skincare, especially the long‐term application of facial creams, leading to an impaired epidermal barrier function, as well as fluoride toothpaste have been suspected as possible causes of POD.^1^, ^3^

A pathogenic role is also being discussed for microbial factors such as infections with *Fusobacterium* spp., *Candida albicans*, and *Demodex folliculorum*. This is in line with the recently published observation, that anti‐COVID‐19 face masks may be another possible predisposing factor as they induce an increase in the temperature of the face with subsequent abnormalities of the microbiota (proliferation of *Staphylococcus epidermidis* and/or *Fusobacterium* spp. and/or *Demodex folliculorum*) and permeability of the skin barrier.^6^ In addition, Dolenc‐Voljc et al.^7^ found significantly higher mite prevalence and mite density values in patients with POD who had prior treatment with topical steroids compared to POD patients without steroid pre‐treatment and to healthy controls (for both *p* < 0.001).

So far, the treatment of POD is mainly based on clinical experience, there are only a few well‐controlled therapy studies.^2^ Therapeutic options include strict avoidance of all topical steroids, cosmetics, and ointments (zero therapy); topical anti‐inflammatory and antimicrobial treatments such as erythromycin, metronidazole, azelaic acid, or pimecrolimus cream; and systemic antibiotics (minocycline, tetracycline, doxycycline). In severe cases, oral retinoids may be considered.^1^, ^2^

Given the potential role of *Demodex folliculorum* in the pathogenesis of perioral dermatitis (POD), similar to rosacea, treatment with topical ivermectin – a macrocyclic lactone disaccharide antiparasitic agent – appears to be a reasonable option. Ivermectin 10 mg/g cream (Soolantra^®^, Galderma Laboratorium GmbH) once daily (QD) is approved for the treatment of inflammatory lesions (papules, pustules) in adult rosacea in the US and Europe since 2014 and 2015, respectively.^8^ The exact mechanism underlying the drug's effectiveness in rosacea remains unclear; however, its therapeutic effect is presumed to result primarily from its anti‐inflammatory and antiparasitic properties.^8^, ^9^, ^10^ Due to these properties, it seems promising to treat POD patients with prior topical steroid therapy (high demodex density) and without prior steroid therapy (normal demodex density) with ivermectin 10 mg/g cream. The decision on the respective therapy was made by the treating physician. The study and data collection complied with all national and local regulations of the ethics committee of the University of Tübingen (project number 856/2023BO2).

There is only one case series with eight patients that has shown therapeutic success in treating POD with topical ivermectin.^9^ These promising results prompted us to conduct a retrospective case series with five female POD‐patients (21–68 years) using off‐label treatment with ivermectin 10 mg/g cream QD as monotherapy or in combination with doxycycline 40 mg modified release capsules. The duration of the disease ranged from 1 month to several weeks, all but one patient had been pre‐treated with various topical agents (Table 1).

**TABLE 1:** Patients baseline characteristics and treatment success with ivermectin 10 mg/g cream QD as monotherapy or in combination with doxycycline 40 mg with modified release.

Pat. no.	Gender	Age (years)	Duration of symptoms	Previous therapy	Demodex mites/ Standardized skin surface biopsy (SSSB)	Current therapy	Application	Course of disease
1	Female	47	1 month	Topical steroid (clobetasol‐propionate)	> 5 Demodex mites/cm^2^ (14 mites in 2 specimens)	Topical ivermectin 1%	0‐0‐1	Good improvement after 8 weeks, complete remission after 16 weeks
2	Female	58	6 months	Topical: steroid, erythromycin, clotrimazole	> 15 Demodex mites/cm^2^ (22 mites in 2 specimens)	Topical ivermectin 1%	0‐0‐1	Good improvement after 8 weeks
3	Female	21	6 weeks	None	Not done	Topical ivermectin 1% + doxycycline 40 mg	0‐0‐1 0‐0‐1	Excellent improvement/complete remission after 8 weeks
4	Female	62	Several months	Topical dimetindene‐maleate, topical steroid	No detection of Demodex mites (3 x)	Topical ivermectin 1%+ doxycycline 40 mg	0‐0‐1 0‐0‐1	No improvement with topical ivermectin after 3 weeks; after addition of doxycycline excellent improvement after 6 weeks, complete remission after 13 weeks
5	Female	68	Not known	Topical steroid, antibiotic ointment	Not done	Topical ivermectin 1% + doxycycline 40 mg	0‐0‐1 0‐0‐1	Excellent improvement/complete remission after 6 weeks

***Note***: Treatment success was evaluated by using the Investigator Global Assessment (IGA) scale with IGA 0 – excellent improvement, IGA 1–2 – good improvement, IGA 3–4 – little/no improvement

All patients were advised to discontinue any topical glucocorticoids, cosmetics and ointments. They responded to the treatment with topical ivermectin very well. Outcome was measured by using the Investigator Global Assessment (IGA) scale (IGA 0 – excellent improvement, IGA 1–2 – good improvement, IGA 3–4 – little/no improvement). Two patients achieved a good improvement (IGA 1–2) after 8 weeks with topical ivermectin alone (Figure 1), two patients an excellent improvement/complete remission (IGA 0) with the initial combination of topical ivermectin 10 mg/g cream QD and doxycycline 40 mg after 6 and 8 weeks (Figure 2), respectively, and one patient after the addition of doxycycline an excellent improvement (IGA 0) after 6 weeks and a complete remission after 13 weeks.

**FIGURE 1:**
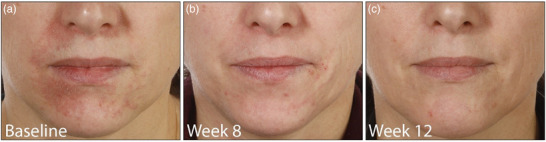
Patient (a) before, (b) after 8 weeks, and (c) after 12 weeks of topical treatment with ivermectin 10 mg/g cream once daily as monotherapy, showing good improvement.

**FIGURE 2:**
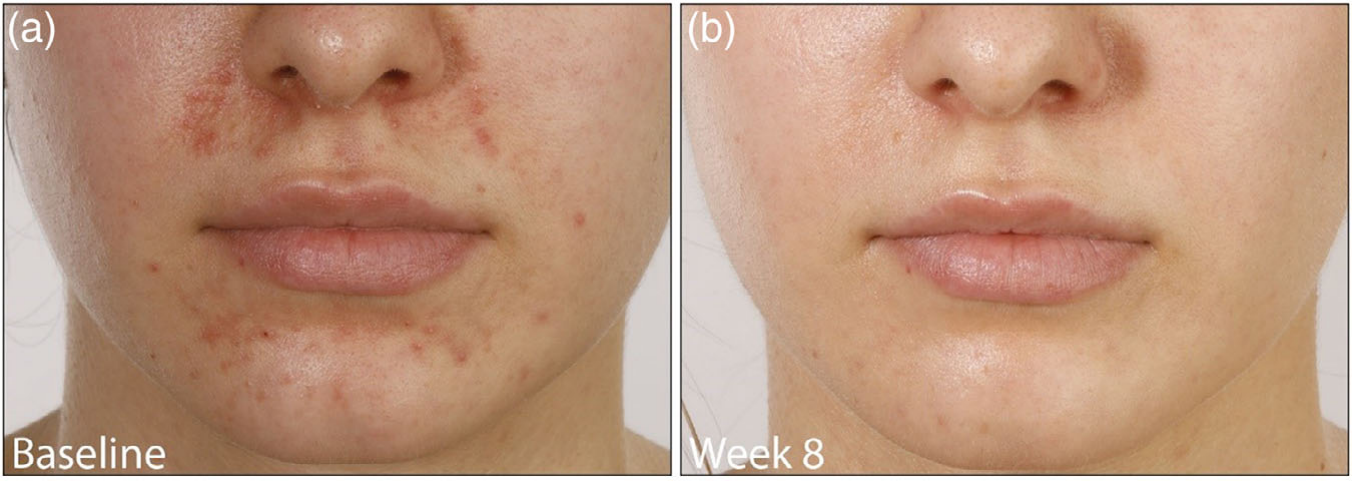
Patient (a) before and (b) after 8 weeks of topical treatment with ivermectin 10 mg/g cream once daily in combination with doxycycline 40 mg modified‐release, showing excellent improvement/complete remission.

In summary, topical ivermectin 10 mg/g cream alone and in combination with oral doxycycline 40 mg was well effective and tolerated for the treatment of POD in patients aged 21–68 years. In particular, the combination of ivermectin 10 mg/g cream + doxycycline 40 mg showed an excellent healing rate and good efficacy in steroid and nonsteroid induced forms of POD. Our results confirm that topical ivermectin is a possible option in adult patients with POD that warrants further study.

The limitations of this study are due to the low number of patients treated and its retrospective character. Large‐scale prospective studies are needed to confirm the results.

## CONFLICT OF INTEREST STATEMENT

M.S. has served as a consultant for AbbVie, Bayer, Galderma, L'Oréal, and UCB in the past three years and has received lecture fees from AbbVie, Galderma, Janssen‐Cilag, Lilly, Mibe, Med Update, and Omnicuris. D.L., G.H., and A.G. declare no conflicts of interest.
